# Intradural paragangliomas in the cauda equina region: a case report and literature review

**DOI:** 10.3389/fonc.2025.1642760

**Published:** 2025-08-07

**Authors:** Jiang-Chun Ma, Xiao-Yong Shi, Hu Sun, Huan Lei, Zhu-Xiao Tang

**Affiliations:** ^1^ Brain Center, Zhejiang Hospital, Hangzhou, Zhejiang, China; ^2^ Department of pathology, Zhejiang Hospital, Hangzhou, Zhejiang, China

**Keywords:** cauda equina paragangliomas, neuroendocrine tumors, diagnose of CEPs, treatment of CEPs, hypertension

## Abstract

**Introduction:**

Cauda Equina Paragangliomas (CEPs) are rare neuroendocrine tumors with an atypical clinical profile. They pose diagnostic and therapeutic challenges due to their varied manifestations and low incidence. This case report aims to contribute to the limited literature on CEPs by detailing the presentation, diagnosis, and surgical management of a new case.

**Case report:**

A 51-year-old female presented with a 20-day history of worsening lumbago and urinary dysfunction. MRI scans revealed a 2cm mass at the L1 vertebral level, leading to the diagnosis of an intraspinal CEP. The patient underwent a successful surgical resection with intraoperative monitoring to manage hemodynamic changes. Postoperative care included hypertension management, and the patient was discharged after a three-week recovery period with a plan for long-term follow-up.

**Conclusion:**

The successful surgical resection of this CEP highlights the importance of precise preoperative imaging and a multidisciplinary approach to management. Despite the rarity of CEPs, this case underscores the feasibility of complete tumor removal and the necessity for long-term monitoring. The evolving landscape of diagnostic and therapeutic tools holds promise for improving outcomes in these rare tumors. Continued research and collaboration are vital for enhancing our understanding and treatment of CEPs.

## Introduction

According to the 2017 classification by the World Health Organization (WHO), paragangliomas are categorized as a type of neuroendocrine tumors that manifest outside the adrenal glands due to their resemblance to pheochromocytoma in terms of the pathological “Zellballen” pattern (1). These tumors have the potential to develop in diverse anatomical sites, such as the head and neck, abdomen, and pelvis. Paragangliomas are further distinguished as either sporadic or hereditary, with the latter being linked to genetic mutations like succinate dehydrogenase (SDHx) gene mutations.

Cauda Equina Paragangliomas (CEPs) are a distinct subset of paragangliomas that display unique pathological and atypical clinical characteristics, despite sharing similarities in pathological structures with other Paragangliomas (PGLs) (2). Additionally, the rare annual incidence of CEPs has contributed to a slower pace of research progress in this area (3).

This case report outlines the clinical presentation, diagnostic evaluation, and treatment approach for a patient diagnosed with an intraspinal cauda equina paraganglioma.

## Case presentation

A 51-year-old female patient presented to our clinic with a twenty-day history of lumbago, reporting an exacerbation of pain characterized by a radiating quality in the left thigh. The pain is notably exacerbated during weight-bearing activities, particularly standing or walking, and worsens when bending over to sweep the floor. In addition, the patient is experiencing progressive urinary and bowel dysfunction. She has no significant medical history, denies a family history of diseases and a history of hypertension, and is not currently taking any medications. The physical examination indicated a transient slight elevation in blood pressure, measuring 170/101 mmHg, and a heart rate of 77 beats per minute. No palpable masses were detected in the neck, and the neurological examination yielded unremarkable findings.

**Figure 1:**
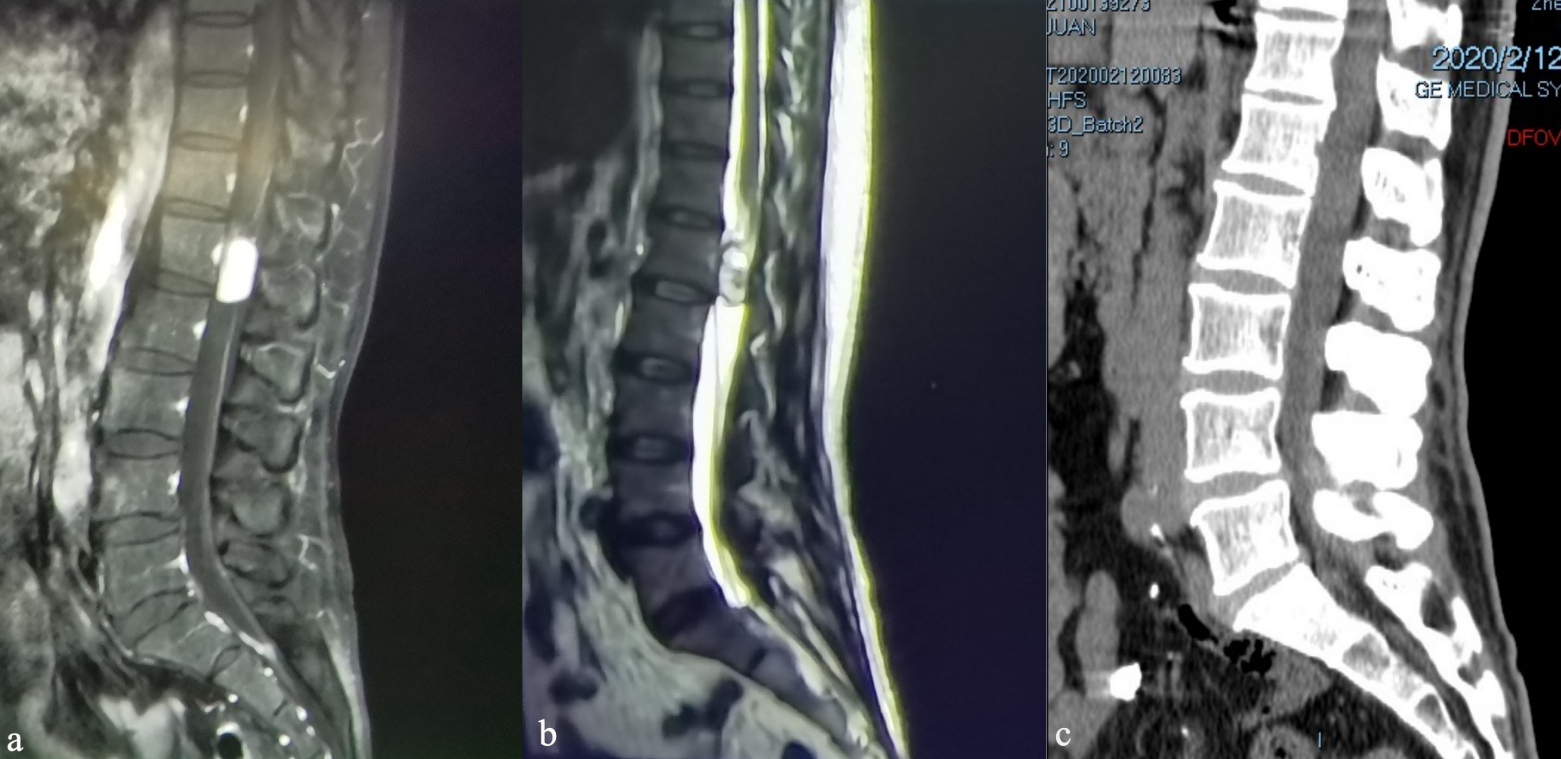
Preoperative magnetic resonance imaging of the lumbar spine, sagittal views: **(a)** sagittal T1-weighted image with contrast (T1WI+C), **(b)** T2WI, **(c)** Computed Tomography.

Additional imaging studies were conducted to explore the etiology of the patient’s symptoms. A magnetic resonance imaging with contrast (MRI+C) scan of the lumbar region identified a 2 cm mass with well-defined margins within the vertebral canal at the L1 level (**Figure 1a**). The displacement of the terminal filament and cauda equina is readily apparent (**Figure 1b**). Furthermore, we have optimized the preoperative lumbar Computed Tomography (CT) protocol to aid in the development of surgical strategies, identification of the surgical bone window, assessment of the positions of the spinous processes and vertebral bodies, localization of tumors, and determination of the necessary size of the bone window for surgical exposure (**Figure 1c**).

## Treatment and outcome

**Figure 2:**
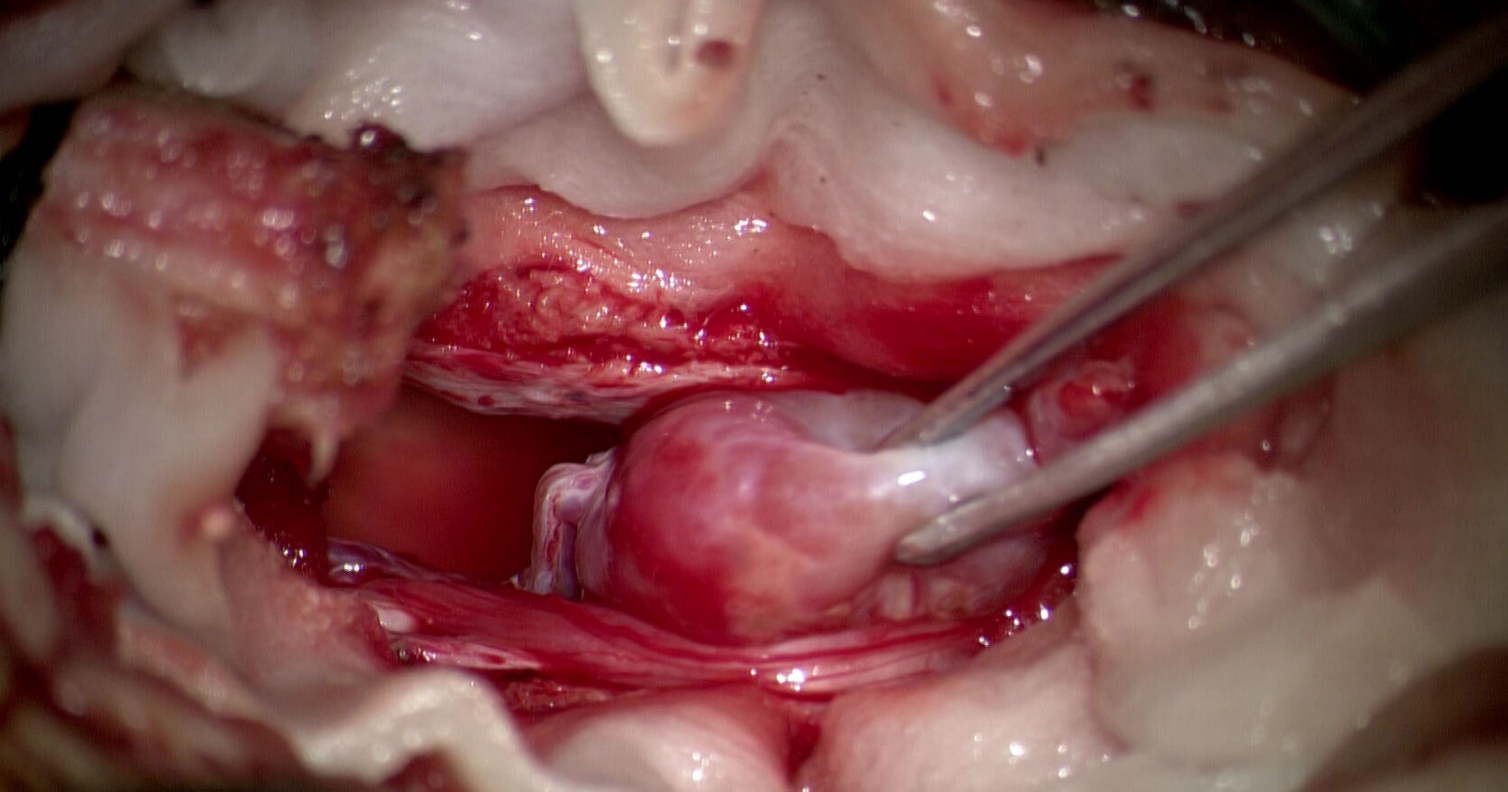
Operative images showing reddish oval tumour with cauda equina and vascular pedicle attached to tumour head.

**Figure 3:**
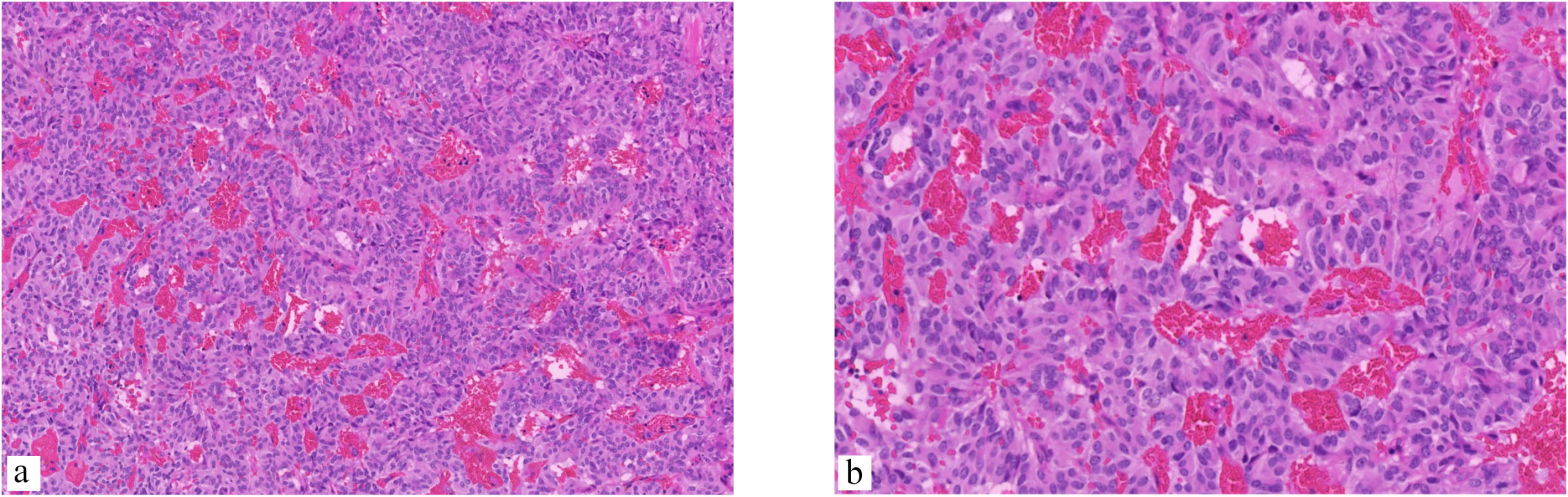
The photomicrograph displays a nest of tumor cells that exhibit a Zellballen pattern. The cells are small and round and are separated by a fine vascular network. **(a)** (Hematoxylin and eosin stain [H&E], ×200 magnification), **(b)** The pseudorosette pattern of tumor cells, characterized by uniformly round to oval nuclei, was accentuated by reticulin staining (H&E, ×400 magnification).

The patient underwent surgical resection of the lumbar mass, which was performed by an experienced lumbar surgeon. Intraoperative monitoring was performed to monitor the patient’s blood pressure and heart rate, which can be affected by manipulation of the tumor. Upon exposure of the surgical field, a grayish-red tumor measuring 2.5cm in length, 1.5cm in width, and 1cm in height was observed, with distinct margins and moderate vascular supply (**Figure 2**). After the complete excision of the tumor, the dura mater was sutured, and the surgical procedure was conducted smoothly. The pathological results are as follows: The photomicrograph displays a nest of tumor cells that exhibit a Zellballen pattern. Well-differentiated neuroendocrine tumor (grade G2). Combined with immunohistochemical results, a primary origin is more likely. Immunohistochemical staining results: CK (Pan) (+). EMA (-). GFAP (-). S-100 (-). CD56 (+). CK20 (focal +). CK7 (-). CgA (+). CEA (-). Vimentin (+). Ki67 (+, 5%). CA199 (-). CDX2 (-). SYN (+). TTF-1 (-). NSE (+). (**Figure 3**).

**Figure 4:**
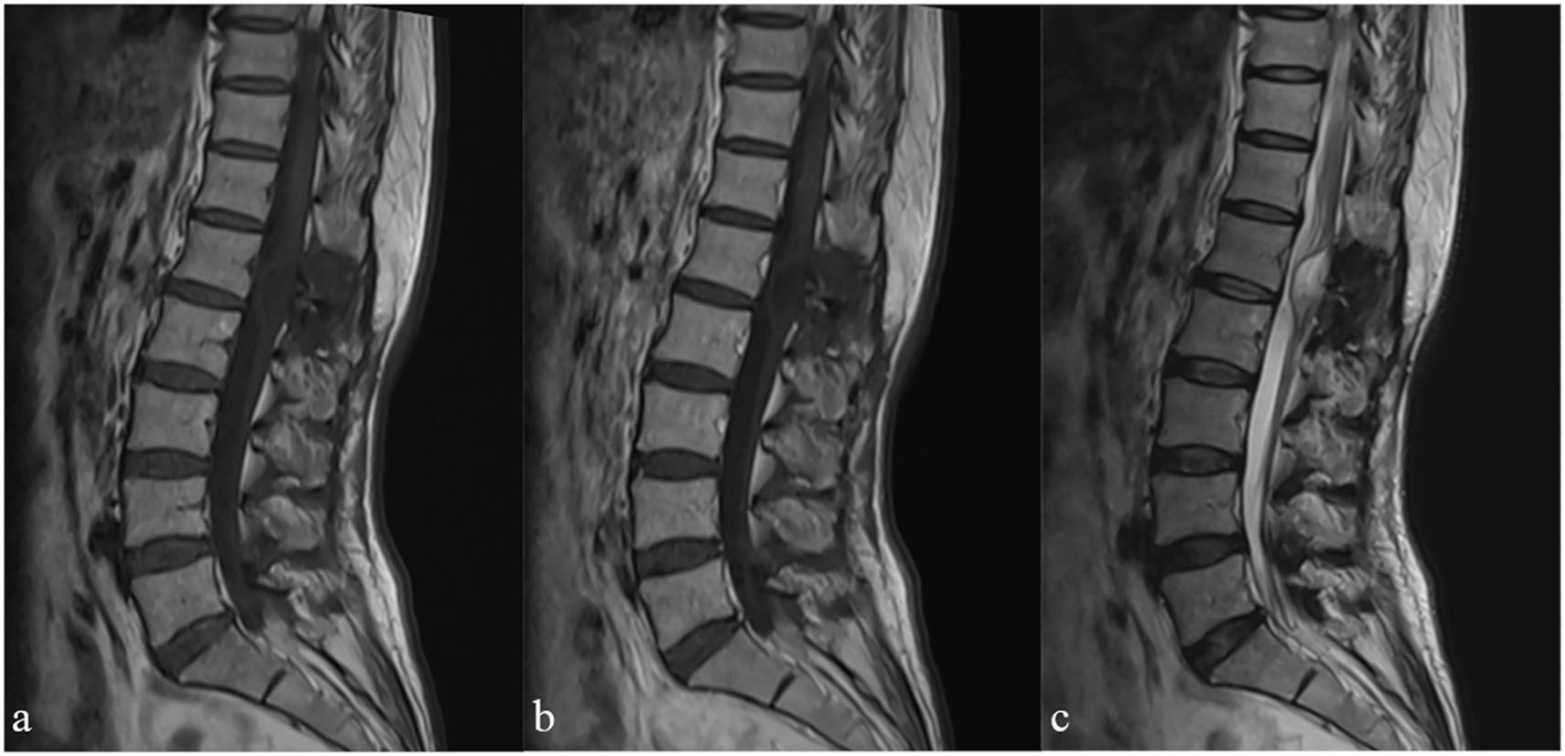
Follow-up magnetic resonance imaging of the lumbar spine, sagittal views: sagittal T1WI **(a)** T1WI+C **(b)** T2WI **(c)**.

Postoperatively, the patient was suffering from persistent headache and hypertension, with the highest blood pressure readings reaching 180/100 mmHg. After the initiation of oral amlodipine besylate for blood pressure management, the patient’s hypertension was well-controlled, and was discharged home after three weeks without oral amlodipine besylate. Follow-up imaging studies were performed in the third year to monitor for recurrence or metastatic disease (**Figure 4**). The patient is currently in good condition, with no neurological deficits, normal bowel and bladder function, and normal blood pressure.

## Review and discussion

Paragangliomas are believed to arise from neural crest cells that differentiate into neuroblasts or chromaffin cells during early embryonic development (4). According to the 2022 WHO classification of tumors, paragangliomas are classified as a neuroendocrine tumor, primarily found in areas with sympathetic or parasympathetic ganglia distribution, including the adrenal medulla (5). Our team conducted a review of case reports on paragangliomas associated with the spinal cord over the past five years and found that the incidence rate of paragangliomas is approximately 0.6 cases per 100,000 individuals, with 90% occurring in the adrenal glands, known as pheochromocytomas, and only 10% occurring extra-adrenal (1, 6). Spinal paragangliomas are even less common, with an incidence rate of about 7 per million (7–10). The average age of diagnosis is 47 years (range 9–77 years), with a male-to-female ratio of approximately 1.54:1 (11, 12). Paolo et al. conducted a study on 334 cases of primary cauda equina paragangliomas, revealing that the majority of tumors were situated in the cauda equina region (81.4%), with the lumbar and lumbosacral regions representing 49.1% and 29% of cases, respectively. Paragangliomas located outside of the cauda equina region were observed in the thoracic spine (11.4%), thoracolumbar region (5.1%), and cervical region (3.6%) (12). In our review of previous cases, we observed that the majority of cases presented with initial symptoms of low back pain (**Table 1**) (13–21). Experienced physicians were often able to promptly arrange spinal magnetic resonance imaging (MRI) for patients, thereby facilitating the identification of the lesion. However, some patients had a history of long-term preoperative follow-up, likely due to the atypical nature of their symptoms. In the case under discussion, the patient’s typical symptoms enabled us to rapidly localize the lesion and achieve total tumor resection. Additionally, the diagnosis of paraganglioma is often not definitive through imaging studies alone. In this case, we mistakenly identified the tumor as a neurofibroma, a misdiagnosis that has also been reported in prior case studies.

**Table 1:** List of reported patients with spinal-related paragangliomas in the last five years.

Article	Age	Gender	Back pain	Bowel/bladder problems	Location of the tumour	Complications (hypertension, cephalalgia.et al.)	Secretory (yes or no)
Ismail Ertan Sevin et al., 2024 (13)	38y	female	Yes	No	L4	No	No
L. Fabbrocini et al., 2024 (14)	59y	female	Yes	Yes	L1-L2	No	No
L. Fabbrocini et al., 2024 (14)	78y	female	Yes	No	L2	No	No
L. Fabbrocini et al., 2024 (14)	35y	male	No	No	L2-L3	No	No
Hristo Popov et al., 2023 (15)	60y	male	Yes	Yes	L3-L4	Yes	Yes
K. Anavi et al., 2023 (16)	23y	female	No	No	T1-T3	Yes	Yes
Dimosthenis Rammos et al., 2022 (17)	47y	male	Yes	No	L3, S2	No	No
David Laville et al., 2021 (18)	56y	female	Yes	No	L2-L3	No	No
Nikolay Konovalov et al., 2022 (19)	55y	female	Yes	Yes	L4	Yes	No
Abolfazl Rahimizadeh et al., 2021 (20)	48y	female	Yes	No	L2	No	No
Frédéric London et al., 2020 (21)	62y	male	Yes	No	L5-S1	No	No

The presence of spinal paragangliomas at different levels of the spinal canal often manifests initially as low back pain in most patients, with some already experiencing symptoms of lower limb radiculopathy, attributed to the mass effect of the tumor (12). The neuroendocrine nature of these tumors, characterized by the secretion of bioamines like adrenaline, noradrenaline, and dopamine, has led researchers to hypothesize that the disease may present with a range of symptoms, including hypertension, palpitations, headaches, and sweating (7, 22). Certain researchers suggest that blood tests measuring levels of somatostatin, serotonin, noradrenaline, adrenaline, dopamine, and homovanillic acid could aid in diagnosing the disease (5, 23). For paragangliomas, catecholamine testing holds particular significance. However, a statistical analysis conducted by Landi et al. suggests that the majority of patients with cauda equina paragangliomas do not present with hypertension, psychomotor distress, or headaches, indicating that hematological tests may not be necessary (10). Some patients may present with preoperative hypertension, which usually alerts physicians to the possibility of paragangliomas. Preoperative MRI imaging suggested a neurofibroma. Intraoperatively, the findings in this case were reminiscent of a neurofibroma. The patient denied the history of hypertension and did not take medication, which made us ignore the preoperative manifestations of increased blood pressure. However, postoperatively, the patient presented headache and increased blood pressure. We initially considered the headache caused by low cranial pressure after the release of cerebrospinal fluid during the operation, which leads to hypertension. This is also why we missed the examination of catecholamines. This case has underscored the importance of a comprehensive assessment, including the evaluation of catecholamines, in similar clinical scenarios. In patient with spinal tumor and hypertension, screening for catecholamine excess is mandatory before surgery to guide intraoperative management and avoid crises.

Magnetic resonance imaging (MRI) is widely acknowledged as a crucial diagnostic tool for spinal canal lesions, with the capability to identify various spinal canal diseases such as neurofibromas, ependymomas and meningiomas, while paragangliomas needed to be distinguished from these tumors (1, 11, 20, 24–26). MRI imaging demonstrates that paragangliomas are extramedullary, subdural spinal tumors characterized by oval or elongated shapes with distinct boundaries. These tumors typically exhibit isodense or hypodense characteristics on T1-weighted images, and hyperdense or heterogeneously dense features on T2-weighted images, often displaying the characteristic “salt and pepper” sign due to the presence of vascular voids within the tumor. Additionally, uniform enhancement of the tumor is commonly observed on T1-enhanced images (27–29), a finding that was corroborated in our specific case.

Pathological examination remains the preferred method for tumor diagnosis, with the 2022 WHO Classification of Endocrine and Neuroendocrine Tumors introducing a new classification for cauda equina paragangliomas as cauda equina neuroendocrine tumors. These tumors can originate from two distinct cell lineages, one epithelial and the other neuroendocrine, with cytokeratin expression serving as a key factor in their classification (5). Previous studies have shown that paragangliomas located in the cauda equina region frequently exhibit pan-cytokeratin (AE1/AE3) expression, whereas paragangliomas in other regions exhibit minimal cytokeratin expression (2, 30). Histologically, a characteristic “Zellballen” pattern is observed on hematoxylin and eosin staining, characterized by spindle-shaped sustentacular cells enveloping chief cells arranged in an alveolar pattern, with an outer layer composed of a fine capillary network (20, 24, 25). This histological feature likely accounts for the encapsulated nature of all tumors. In contrast to Ependymoma, paragangliomas located in the cauda equina region do not exhibit expression of GFAP and EMA. Instead, the presence of neuroendocrine markers such as CgA, Syn, NSE, CD56, and S-100 can be utilized as distinguishing diagnostic factors. Furthermore, differentiation between hemangioblastoma and carcinoid tumors can be accomplished through the examination of Syn and S-100, respectively (30). Transcription factors SATA3, CDX2, and TTF-1 are typically absent in CEPs, aligning with the characteristics observed in the case under consideration (3, 31).

In recent years, advancements in sequencing technology have led to significant improvements in genetic and epigenetic research on CEPs (2, 3, 5, 31), which is essential for understanding the classification and origins of these entities. The SDHx family, a well-studied group of tumor suppressors, may have a significant impact on the metastatic behavior of paragangliomas and pheochromocytomas, with SDHB already incorporated into certain tumor scoring systems (5, 32). Additional genetic alterations, such as telomerase activation, ATRX mutations, high mutational burden, and MAML3 gene fusions, require further validation (33, 34). The genetic profiles of CEPs exhibit significant divergence from paragangliomas located in other anatomical regions, with several retrospective studies reporting an absence of SDHx mutations in CEPs (2, 3, 24, 31, 35, 36). Additionally, investigations into methylation patterns conducted by Ramani et al. suggest that CEPs may not share homology with other epithelial neuroendocrine tumors, as evidenced by distinct epigenetic methylation clustering profiles (3).

Surgical resection is the recommended primary treatment for localized paragangliomas of the cauda equina. Intraoperative monitoring is crucial to prevent potentially fatal hypertensive crises (12, 25). Beta-blockers may be utilized before and after surgery to manage blood pressure and heart rate (23). We recommend that MRI examination will be necessary every 3–5 years. Long-term surveillance is essential to detect any recurrence or metastasis, especially in cases of hereditary paragangliomas (9). The necessity of adjuvant radiotherapy and chemotherapy following surgery remains a contentious issue in the medical community (37). Empirical evidence from clinical practice supports the efficacy of preventive radiotherapy for patients with unresectable tumors (29). The coordination of a multidisciplinary team of specialists is imperative for the comprehensive management of these uncommon malignancies.

The current advancements in high-throughput sequencing and liquid biopsy represent promising new technologies that are anticipated to facilitate early diagnosis of rare conditions such as CEPs. These innovations also offer significant assistance in the management and prognostic prediction of such diseases. The case study we present augments the already limited number of reported cases of paragangliomas in the cauda equina region. However, considering the patient’s economic circumstances, we have not pursued further genetic testing for this case. In future research endeavors, the application of these emerging technologies is expected to provide researchers with novel perspectives for the investigation of conditions like CEPs.

## Data Availability

The original contributions presented in the study are included in the article/supplementary material. Further inquiries can be directed to the corresponding author.
